# Genomic Analysis Reveals Novel Specific Metastatic Mutations in Chinese Clear Cell Renal Cell Carcinoma

**DOI:** 10.1155/2020/2495157

**Published:** 2020-09-30

**Authors:** Hui Meng, Xuewen Jiang, Jianfeng Cui, Gang Yin, Benkang Shi, Qi Liu, He Xuan, Yu Wang

**Affiliations:** ^1^Department of Urology, Qilu Hospital of Shandong University, 107 Jinan Culture Road, Jinan, 250012 Shandong, China; ^2^Life Healthcare Medical Laboratory Co., Ltd., Hangzhou, 310052 Zhejiang, China; ^3^Reproductive Medicine Center, Department of Obstetrics and Gynecology, Qilu Hospital of Shandong University, 107 Jinan Culture Road, Jinan, 250012 Shandong, China

## Abstract

Clear cell renal cell carcinoma (ccRCC) accounts for more than 75% of renal cell carcinoma. Nearly 25% of ccRCC patients were diagnosed with metastasis. Though the genomic profile of ccRCC has been widely studied, the difference between localized and metastatic ccRCC was not clarified. Primary tumor samples and matched whole blood were collected from 106 sporadic patients diagnosed with renal clear cell carcinoma at Qilu Hospital of Shandong University from January 2017 to November 2019, and 17 of them were diagnosed with metastasis. A hybridization capture-based next-generation sequencing of 618 cancer-related genes was performed to investigate the somatic and germline variants, tumor mutation burden (TMB), and microsatellite instability (MSI). Five genes with significantly different prevalence were identified in the metastatic group, especially *TOP1* (17.65% vs. 0%) and *SNCAIP* (17.65% vs. 0%). The altered frequency of *PBRM1* (0% vs. 27%) and *BAP1* (24% vs. 10%) differed between the metastatic and nonmetastatic groups, which may relate to the prognosis. Of these 106 patients, 42 patients (39.62%) had at least one alteration in DNA damage repair (DDR) genes, including 58.82% of metastatic ccRCC patients and 35.96% of ccRCC patients without metastasis. Ten pathogenic or likely pathogenic (P/LP) variants were identified in 11 sporadic clear cell renal cell carcinoma patients (10.38%), including rarely reported *ATM* (n=1), *MUTYH* (n=1), *NBN* (n=1), *RAD51D* (n=1), and *BRCA2* (n=1). No significant difference in the ratio of P/LP variant carriers or TMB was identified between the metastatic and nonmetastatic groups. We found a unique genomic feature of Chinese metastatic ccRCC patients with a higher prevalence of alterations in DDR, *TOP1*, and *SNCAIP.* Further investigated studies and drug development are needed in the future.

## 1. Introduction

Renal cell carcinoma (RCC) is the seventh most common cancer in men and the ninth most common cancer in women, accounting for 2 to 3 percent of all adult malignancies. The estimated incidence and mortality of RCC in China in 2015 were 66,800 and 23,400, respectively [1]. RCC comprises more than 10 histological and molecular subtypes, with differences in the histological pattern, clinical course, and genomic feature. Of these types, clear cell renal cell carcinoma (ccRCC) accounts for the most proportion (75%-80%) and has a worse prognosis [2]. To date, surgery, especially radical nephrectomy, is still the most effective treatment for localized ccRCC patients. However, nearly 25% of RCC patients were diagnosed with metastasis and unable to take surgery to remove the tumor [3]. Besides, RCC is commonly resistant to chemotherapy and radiotherapy, so the 5-year survival was poor. A clear understanding of the genomic features of metastatic ccRCC will help us to select personalized treatments and develop new effective drugs.

With the widespread next-generation sequencing technology, the genomic landscape of ccRCC has been revealed by many studies [4]. ccRCC was mainly characterized by the loss of chromosome 3p, and alterations of genes involved in this location, especially *VHL*, *SETD2*, *PBRM1*, and *BAP1*, are suggested as the driver events of ccRCC [5]. Research on the genomic feature of Chinese ccRCC patients is deficient, and limited results showed a similar driver role of *VHL*, *SETD2*, *PBRM1*, and *BAP1* genes and unique genes involved in the ubiquitin-mediated proteolysis pathway [6]. A comparison of genetic differences between localized and metastatic ccRCC has not been well depicted yet. A significant higher mutated frequency of *TP53* was found in the metastatic ccRCC, but no difference in other genes or tumor mutation burden was found [7].

We conducted a genomic study to determine the difference in the gene prevalence between Chinese nonmetastatic and metastatic ccRCC patients and discovered potential related to metastasis.

## 2. Materials and Methods

### 2.1. Biospecimen Collection and Clinical Data

Primary tumor samples and matched whole blood were collected from 106 sporadic patients diagnosed with renal clear cell carcinoma at Qilu Hospital of Shandong University from January 2017 to November 2019. 17 of them were diagnosed with metastasis, and the rest was localized ccRCC. Written informed consent was obtained from each patient for collection and further publication of their clinical details and tumor genomic profiles. All enrolled patients did not receive any neoadjuvant treatment before sample collection, including chemotherapy, anti-VEGFR or mTOR therapy, and immunotherapy. All tumor samples were pathologically reviewed to have at least 60% tumor cells. Clinical data were collected, including patient ID, gender, age, and metastatic status at diagnosis (Supplementary Table S1).

### 2.2. Target Next-Generation Sequencing

For the matched blood and tumor samples, germline DNA (gDNA) was isolated using the DNeasy Blood & Tissue Kit (Qiagen, 69504) according to the manufacturer's instruction. 100 ng of gDNA was sheared with a Covaris E210 system (Covaris, Inc.) to target fragment sizes of 200 bp. We performed library preparation for tumor gDNA and matched germline gDNA using the Accel-NGS 2S DNA Library Kit (Swift Biosciences, Inc.) and target enrichment using the xGen Lockdown Probe kit (IDT, Inc.). The custom xGen Lockdown Probe was synthesized by IDT, Inc. for the exons and parts of introns of 618 genes of interest (Supplementary Table S2). Samples underwent paired-end sequencing on an Illumina Nextseq CN500 platform (Illumina Inc.) with a 150 bp read length and mean coverage of 1000x at a 95% capture rate and 40% dup rate.

### 2.3. Data Analysis

Raw sequencing data were aligned to the reference human genome (UCSC hg19) through Burrows-Wheeler Aligner and producing a binary alignment/map (BAM) file. After the duplicate removal and local realignment, the Genome Analysis Toolkit (GATK) was used for single-nucleotide variation (SNV) and short insertion/deletion (indel) calling. Variants were annotated using the ANNOVAR software tool. Variants identified in gDNA from buffy coat fraction aliquots with allele fraction (AF) beyond 25% were determined as germline variants. The interpretation of germline variants followed the standards and guidelines of the American College of Medical Genetics and Genomics and the Association for Molecular Pathology (ACMG/AMP) and was independently reviewed by two genetic consultants [8]. Somatic variants with AF beyond 1% were generated from each tumor gDNA by removing the germline variants and further annotated according to the Catalog of Somatic Mutations in Cancer (COSMIC) database. The functional classification of each somatic mutation followed the interpretation and reporting standards and guidelines recommended by the Association for Molecular Pathology, American Society of Clinical Oncology, and College of American Pathologists (ASCO/CAP) [9]. The tumor mutation burden of each sample was calculated according to a published and widely applied method [10].

### 2.4. Statistical Analysis

Differential mutation analysis was performed using the Chi-squared test or Fisher exact test under a dominant model. Two-sided *P* values less than 0.05 were considered to be statistically significant. All analyses were performed using SPSS 25.0 software.

## 3. Results

### 3.1. Somatic Mutation Landscape of ccRCC with/without Metastasis

The clinical data of 106 enrolled ccRCC patients of primary kidney tissues (comprising of 89 localized and 17 metastatic ccRCC) is shown in Table 1.

**Figure 1:**
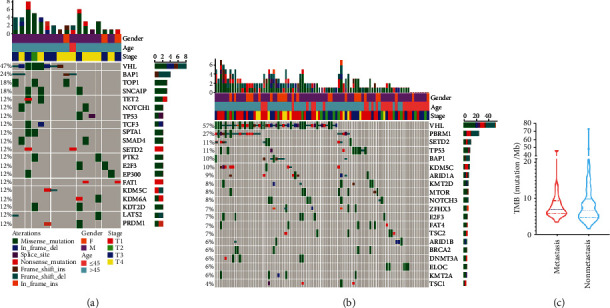
The genomic landscape of somatic mutations in ccRCC with/without metastasis. (a) Oncoprint illustrations of somatic alterations in metastatic ccRCC by gene frequency. (b) Oncoprint illustrations of somatic alterations in nonmetastatic ccRCC by gene frequency. (c) The difference in tumor mutation burden (TMB) between the metastasis and nonmetastasis groups.

The prevalence of somatic altered genes in metastatic and nonmetastatic ccRCC patients is shown in Figure 1. We found that the most frequently altered genes in metastatic ccRCC were *VHL* (47%), *BAP1* (24%), *TOP1* (18%), and *SNCAIP* (18%) in metastatic patients (Figure 1(a)). The most altered genes in nonmetastatic ccRCC were *VHL* (57%), *PBRM1* (27%), *SETD2* (11%), and *TP53* (11%), respectively (Figure 1(b)). Meanwhile, the median TMB of the metastatic and nonmetastatic ccRCC patients was 7.56 and 6.67 mutations/Mb, respectively, with no significant difference (Figure 1(c)). No microsatellite instability-high (MSI-H) tumor was identified in the 106 ccRCC samples.

**Figure 2:**
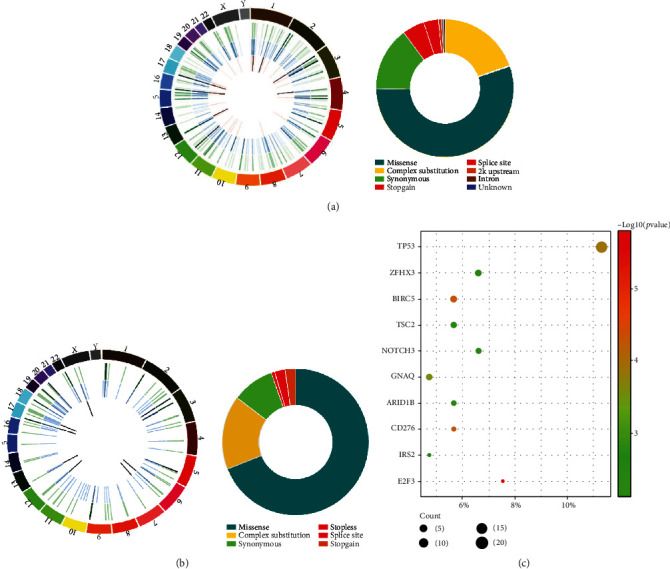
Comparison of somatic mutation patterns in ccRCC with/without metastasis on the chromosome and the frequency of differential mutation genes in the TCGA ccRCC cohort. The Circos plot and pie plot display gene mutational patterns on the chromosome and frequency of every variant (a) in the nonmetastatic group and (b) in the metastatic group. (c) The diagram displays mutational gene frequencies of the total 106 ccRCC patients (Fisher's exact test), compared with the TCGA data.

We used the Circos plot to display the gene mutations on the chromosome and found that the somatic mutations were widely distributed in the nonmetastatic ccRCC but relatively concentrated in the metastatic ccRCC in contrast. The four mutational patterns with the highest frequency in ccRCC patients were missense (metastatic versus nonmetastatic group, 68.99% vs. 54.91%), complex substitution (16.28% vs. 19.85%), synonymous (9.30% vs. 15.03%), and splice site (2.33% vs. 5.20%) (Figures 2(a) and 2(b)). The prevalence of altered genes of the total of 106 ccRCC patients was compared with The Cancer Genome Atlas (TCGA) data. Four genes with both high mutation frequency and low *P* value were identified, including *ZFHX3* (our cohort versus TCGA, 6.60% vs. 1.17%), *NOTH3* (6.60% vs. 1.41%), *ARID1B* (5.66% vs. 0.94%), and *TSC2* (5.66% vs. 0.94%) (Figure 2(c)). Notably, a significantly higher frequency of altered *E2F3* was identified in our cohort (7.55% vs. 0%) (Supplementary Table S3).

### 3.2. Differences in the Prevalence of Altered Genes between ccRCC with or without Metastasis

**Figure 3:**
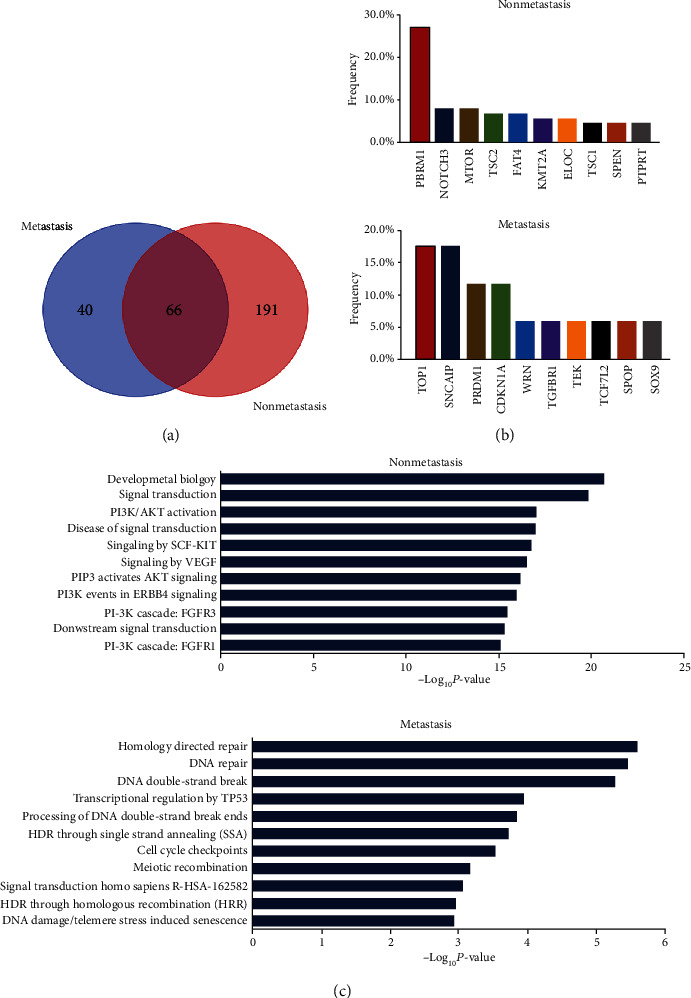
Analysis of genes with different prevalence in the nonmetastatic and metastatic groups. (a) Venn diagram illustrated the overlap and independently mutated genes between the nonmetastatic and metastatic groups. (b) The frequency of genes with different prevalence in the nonmetastatic (bottom) and metastatic (top) groups. (c) Reactome pathway analysis of mutated genes with different prevalence in the nonmetastatic (bottom) and metastatic (top) groups.

Next, we investigated the different frequencies of altered genes between the metastatic and nonmetastatic groups (Figure 3(a)). The metastatic and nonmetastatic groups harbored 191 and 40 genes with different mutated frequencies, respectively. The top ten unique mutated genes involved in the nonmetastatic cohort (Figure 3(b)) were *PBRM1*, *NOTCH3*, *MTOR*, *TSC2*, *FAT4*, *KMT2A*, *ELOC*, *TSC1*, *SPEN*, and *PTPRT.* On the contrary, a different prevalence of *TOP1*, *SNCAIP*, *PRDM1*, *CDKN1A*, *WRN*, *TGFBR1*, *TEK*, *TCF7L2*, *SPOP*, and *SOX9* was involved in the metastatic cohort genes (Figure 3(b) and Supplementary Table S4). Reactome pathway analysis of the genes with different prevalence showed that the nonmetastatic group was characterized by gene sets associated with developmental biology, signal transduction signaling, PI3K/AKT activation, and cancer classic signaling pathways (Figure 3(c)), while the metastatic group was characterized by gene sets associated with homology recombination repair, DNA repair, and DNA double-strand break signaling pathway (Figure 3(c)).

### 3.3. Details on Genes with Different Prevalence in ccRCC with or without Metastasis

**Figure 4:**
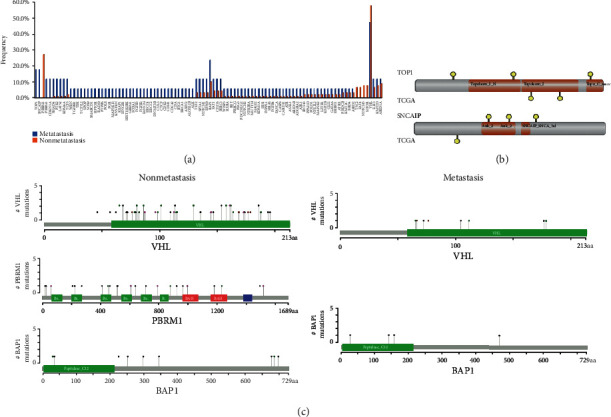
Analysis of differential mutation genes. (a) 101 mutated genes of different frequencies between the metastatic and nonmetastatic groups. (b) The location of identified *TOP1* and *SNCAIP* variants. (c) The distribution of identified *VHL*, *PBRM1*, and *BAP1* variants in the nonmetastatic (left) and metastatic (right) groups. The grey bar represents the entire protein marked with an amino acid number. The height of the grey line indicates the number of a specific mutation, and the colored circle on the grey line represents the corresponding mutation types. Green: missense; black: truncating; brown: inframe indels; purple: other.

Specifically, we identified 101 genes with different prevalence between the metastatic and nonmetastatic cohorts. Lollipop plots showed the distribution of *VHL*, *PBRM1*, and *BAP1* mutations, and truncating was the most common mutation types in three genes, followed by missense and inframe indels (Figure 4(a)). Notably, *TOP1* and *SNCAIP* variants were only identified in the metastatic group (*P* = 0.0035, Table 2). The distribution of *TOP1* and *SNCAIP* variants is illustrated in Figure 4(b). On the contrary, *PBRM1* was only mutated in the nonmetastatic group (Figure 4(c)). No significant difference was found in VHL and BAP1 between the two groups.

### 3.4. Somatic Alterations in DNA Damage Repair (DDR) Pathway

**Figure 5:**
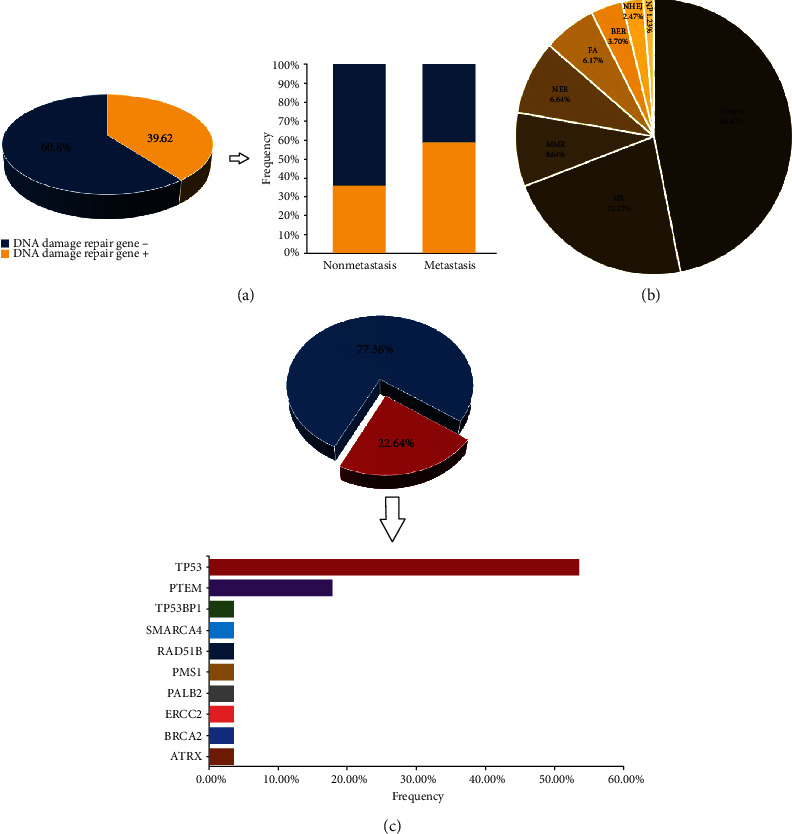
Analysis of somatic alterations in the DDR pathway. (a) The frequency of DDR mutations across ccRCC patients with or without metastasis. (b) The frequency of specific mutated pathway in DDR. (c) Frequency of mutated DDR genes with known deleterious variants. HR: homology recombination; MMR: mismatch repair; NER: nucleotide excision repair; FA: Fanconi anemia; BER: base excision repair; NHEJ: nonhomology end joining; NP: nucleotide pool maintenance.

Of these 106 patients, 42 patients (39.62%) had at least one alteration in DDR genes, including 58.82% of metastatic ccRCC patients (10/17) and 35.96% of ccRCC patients without metastasis (32/89) (Figure 5(a)). The most common mutational pathway was the homology recombination (HR) repair pathway (22.22%), followed by the mismatch repair (MMR) pathway (8.64%, Figure 5(b)).

Among the DDR gene alterations, 22.64% of them were pathogenic. Specifically, the DDR genes with the most known or likely deleterious variants were *TP53* and *PTEN* (Figure 5(c)).

### 3.5. Germline Mutations in ccRCC

Ten pathogenic or likely pathogenic (P/LP) variants were identified in 11 sporadic clear cell renal cell carcinoma patients (10.38%). None of the P/LP carriers was with bilateral or multiple renal masses (Table 3). Three patients were with a family cancer history, but none had familial renal cell cancer. The mean age and median age at diagnosis of identified P/LP carriers were 50.72 and 50 years, respectively. Only 4 P/LP carriers (36.36%) were diagnosed below 46 years old. There were no significant differences in the age at diagnosis between P/LP carriers and noncarriers. Except *VHL* and *FH* genes, which have been widely identified in hereditary kidney cancer, we found P/LP variants in rarely reported *ATM* (*n* = 2), *MUTYH* (*n* = 2), *NBN* (*n* = 2), *RAD51D* (*n* = 1), and *BRCA2* (*n* = 1) genes in our cohort. No significant difference in the P/LP variant carriers' ratio was identified between the metastatic and nonmetastatic groups.

^#^Patients 022, 024, and 092 were ccRCC with metastasis. ^∗^Two sporadic patients had the same *ATM* variants.

## 4. Discussion

The genomic differences between Chinese metastatic and nonmetastatic ccRCC have not been revealed. Compared with TCGA, a higher prevalence of *ZFHX3*, *NOTH3*, *ARID1B*, *TSC2*, and *E2F3* was identified in our cohort, suggesting a difference in the genomic feature between Chinese and Caucasian ccRCC patients. A past research found a prognostic value of E2f3 expression in ccRCC, which was significantly associated with tumor size, metastasis, lymph node metastasis, and tumor stage [11].

The genomic profile of ccRCC has been widely studied and is mainly characterized by the loss of chromosome 3p [5]. Alterations of genes involved in this location, especially *VHL*, *SETD2*, *PBRM1*, and *BAP1*, are suggested as the driver events of ccRCC (Supplementary Table S5). Interestingly, we only identified *PBRM1* variants in the nonmetastatic ccRCC group (27%); on the contrary, a higher *BAP1* prevalence in the metastatic ccRCC group (24% vs. 10%). *BAP1* mutation is associated with worse survival in ccRCC, and *PBRM1*-mutated patients had a favorable survival instead [12]. Differences in the prevalence of these two genes between localized and metastatic ccRCC may contribute to the different prognosis effects on survival. Meanwhile, *PBRM1* alterations may correlate with the sensitivity of immune checkpoint therapy in ccRCC, though there are controversial results on its immunogenic effects [13, 14].

We only identified *TOP1* and *SNCAIP* alterations in the metastatic ccRCC, which was not reported before. *TOP1* encodes topoisomerase I (Top1), which is involved in the process of DNA replication and chromosomal recombination. As depicted by the immunochemistry analysis, Top1 expression was elevated in 23.5% of RCC, and increased expression was associated with a higher grade (grades 3 and 4) [15]. Only 0.7% of ccRCC patients had *TOP1* alterations in the TCGA database, and all alterations were missense both in TCGA and our cohort. Camptothecin and related drugs, including irinotecan, were designed to inhibit tumor cell proliferation by trapping Top1 on the DNA to stop the DNA replication process. However, the *TOP1* alterations we identified (including p.His399Arg, p.Thr154Lys, and p.Gln713Glu) were not function clarified yet, so the potential response to the camptothecin-related drugs could not be predicted. The other unique marker of metastasis we identified was *SNCAIP*, a gene encoded synuclein alpha interacting protein, which was mainly related to Parkinson disease. Due to the deficiency of research, the oncogenic or tumor suppressor role of *SNCAIP* was not well depicted [16]. Promoter hypermethylation of *SNCAIP* could serve as a marker for colorectal cancer identification [17]. A previous study revealed that *SNCAIP* mutations were uniquely found in the diabetic group in pancreatic cancer, mainly involved in immune-related pathways [18]. In bladder cancer, *SNCAIP* was dysregulated and identified as a hub gene for predicting disease progression and prognosis [19]. Meanwhile, *SNCAIP* was rarely mutated (0.44%) but highly amplified (11.61%) in ccRCC in the TCGA database. So, it is worthy of additional studies on *SNCAIP* function and potentially drug development in ccRCC. We also identified *CDKN1A* alterations in the metastatic groups, and loss of *CDKN1A* had been proved to be an unfavorable predictor of prognosis in chromophobe renal cell carcinoma patients [20].

The application of immune checkpoint inhibitors (ICIs) in ccRCC has been rapidly progressing in the past years, so the potential response biomarkers are arresting significant interests. The primary known molecular biomarkers for ICIs were MSI-H and TMB, respectively. Previous research had found that MSI-H was rare (0.6%) in RCC, which was also proved in our cohort as no MSI-H tumor was identified in the 106 ccRCC [21]. Research based on unmatched primary and metastatic non-small-cell lung carcinoma found a higher TMB trend in metastatic tumors [22]. However, no significant difference was found between metastatic and nonmetastatic tumors in our study. Limited by the sample sizes, we suggested that further research would be needed to reveal the difference in TMB between metastatic and nonmetastatic ccRCC. Furthermore, recent research found a correlation between DDR gene alterations and ICIs [23]. A retrospective analysis found that 5% and 16% ccRCC patients had germline and tumor variants in DDR genes, respectively. Meanwhile, oncogenic DDR gene variants were associated with better overall survival in immune-oncology but not in the tyrosine kinase inhibitor cohort [24]. A relatively higher incidence of DDR mutation was identified in the metastatic groups (58.82% versus 35.96%) in our research. Whether metastatic ccRCC patients with DDR mutation would benefit from ICIs, PARP inhibitor, or platinum needed further investigation.

Hereditary kidney cancer was estimated to account for 5%-8% of newly diagnosed kidney cancer. A recently published study found that 9.5% of Chinese sporadic early-onset (below 46 years old at diagnosis) had P/LP variants in 10 genes and a significant correlation to family cancer history in second-degree relatives [25]. In our cohort, 10.38% of sporadic ccRCC patients had P/LP germline variants, and the main reason for the higher frequency may be because of the more widely analyzed genes involved. Notably, 81.82% (9/11) P/LP variants were in genes related to the DDR repair pathway. Of patients with P/LP variants, 7 (63.64%) would not have been referred for genetic evaluation according to NCCN and Chinese kidney cancer guidelines.

## 5. Conclusions

This present study demonstrated the genetic landscape of Chinese nonmetastatic and metastatic ccRCC patients. Different prevalence of genes, especially *TOP1* and *SNCAIP*, was suggested to associate with metastasis. On the contrary, *PBRM1* was correlated with nonmetastatic disease. A higher incidence of DNA damage repair gene alteration was identified in the metastatic disease. Further development of target drugs on *TOP1*, *SNCAIP*, and DDR might be useful for Chinese metastatic ccRCC patients.

## Figures and Tables

**Table 1:** Clinical characteristics of 106 ccRCC patients.

Characteristics	Metastatic group (*n* = 17)	Nonmetastatic group (*n* = 89)
Age		
Median	57 (34-74)	55 (25-86)
Sex		
Male	14	64
Female	3	25
Stage		
I	0	38
II	1	13
III	7	27
IV	9	11

**Table 2:** Genes with different mutated frequencies in ccRCC with or without metastasis.

Gene	Metastasis (*n* = 17)		Nonmetastasis (*n* = 89)		*P* value
	Samples with mutation	Samples without mutation	Samples with mutation	Samples without mutation	
*TOP1*	3	14	0	89	0.004
*SNCAIP*	3	14	0	89	0.004
*PBRM1*	0	17	24	65	0.011
*PRDM1*	2	15	0	89	0.024
*CDKN1A*	2	15	0	89	0.024
*SMAD4*	2	15	1	88	0.066
*PTK2*	2	15	1	88	0.066
*LATS2*	2	15	1	88	0.066
*KDM6A*	2	15	1	88	0.066
*TCF3*	2	15	2	87	0.120
*WRN*	1	16	0	89	0.160
*TGFBR1*	1	16	0	89	0.160
*TEK*	1	16	0	89	0.160
*TCF7L2*	1	16	0	89	0.160
*SPOP*	1	16	0	89	0.160

**Table 3:** Details of pathogenic or likely pathogenic variant carriers.

ID^#^	Age	Family history	Gene	Exon	Nucleotide change	Amino acid change
005	50	No	*RAD51D*	4	c.271_272insTA	p.Lys91fs
016	45	No	*MUTYH*	2	c.55C>T	p.Arg19Ter
022	34	Grandmother	*VHL*	2	c.345C>G	p.His115Gln
024	54	Father	*NBN*	5	c.499_500insT	p.Cys167fs
037	37	No	*MUTYH*	13	c.1214C>T	p.Pro405Leu
041	66	No	*BRCA2*	11	c.3098_3099delAT	p.Asp1033fs
056	67	No	*NBN*	14	c.2167delC	p.Leu723Ter
058^∗^	38	No	*ATM*	10	c.1402_1403delAA	p.Lys468fs
069	65	Brother	*RAD50*	12	c.1821_1822insA	p.Lys608fs
076^∗^	47	No	*ATM*	10	c.1402_1403delAA	p.Lys468fs
092	55	No	*FH*	3	c.301C>T	p.Arg101Ter

## Data Availability

The data used to support the findings of this study are available from the corresponding author upon request.

## Supplementary Materials

Supplementary materials Clinical information of metastasis (Supplementary Table S1), targeted sequencing genes in the article (Supplementary Table S2), differential mutations between our data and TCGA dataset (Supplementary Table S3), variant allele fraction of the mutations (Supplementary Table S4), and driver genes (Supplementary Table S5) are provided in supplementary tables.
